# The Back2School modular cognitive behavioral intervention for youths with problematic school absenteeism: study protocol for a randomized controlled trial

**DOI:** 10.1186/s13063-018-3124-3

**Published:** 2019-01-08

**Authors:** Mikael Thastum, Daniel Bach Johnsen, Wendy K. Silverman, Pia Jeppesen, David A. Heyne, Johanne Jeppesen Lomholt

**Affiliations:** 1 Department of Psychology and Behavioral Sciences, Aarhus University, Aarhus, Denmark; 2 Yale Child Study Center, New Haven, CT USA; 3 Institute for Clinical Medicine, Faculty of Health and Medical Sciences, University of Copenhagen, Copenhagen, Denmark; 4 Child and Adolescent Mental Health Center, Mental Health Services of the Capital Region of Denmark, Copenhagen, Denmark; 5 Institute of Psychology, Faculty of Social and Behavioral Sciences, Leiden University, Leiden, The Netherlands; 6 TrygFonden’s Center for Child Research, Aarhus University, Aarhus, Denmark

**Keywords:** School absenteeism, Cognitive behavioral therapy, Transdiagnostic, Randomized controlled trial

## Abstract

**Background:**

School absenteeism (SA) is associated with anxiety, depression, and disruptive behavior. It is a risk factor for academic difficulties and school dropout, which predict problems in adulthood such as social, work-related, and health problems. The main goal of this study is to examine the initial effectiveness of a modular transdiagnostic cognitive behavioral therapy (CBT) intervention (Back2School) for increasing school attendance and decreasing psychological problems, relative to a comparator control arm (treatment as usual [TAU]).

**Methods/design:**

One hundred sixty children, aged 7 to 16 years, will be randomly assigned to either Back2School or TAU. The design is a two (Back2School and TAU) by four (preassessment [T1], postassessment [T2], and 3-month [T3] and 1-year [T4] assessments) mixed between-within design. The primary outcome is school attendance based on daily registration. Secondary outcomes pertain to youth psychosocial functioning, quality of life, bullying, self-efficacy, and teacher-parent collaboration. These secondary outcomes are measured via youth, parent, and teacher reports.

**Discussion:**

This study will provide critically needed empirical evidence on the initial effectiveness of a manualized treatment program for youth with SA. If the intervention is found to be effective, the program can be further implemented and tested in a larger school health effectiveness trial.

**Trial registration:**

ClinicalTrials.gov, NCT03459677. Retrospectively registered on 9 March 2018.

**Electronic supplementary material:**

The online version of this article (10.1186/s13063-018-3124-3) contains supplementary material, which is available to authorized users.

## Background

School is a central context for youth development [1], playing a major role in teaching youth the values of society and preparing them for adult life. Absence from this central context may be precipitated and/or maintained by anxiety, depression, and disruptive behavior [2–4]. School absenteeism (SA) is also a risk factor for academic difficulties and school dropout, all of which are additional predictors of social, work-related, and health problems in adulthood [5–7]. Each day of absence has been shown to have an impact on academic achievement [8]. For Danish schoolchildren, significant negative associations exist between SA on the one hand and school grades, the likelihood of starting secondary education, and the likelihood of completing secondary education on the other hand. Academic and social well-being are significantly lower when there are high rates of SA [9].

In Denmark, the mean rate of SA is 5.6%, amounting to approximately 11 days during a school year [9]. Almost all children are absent from school a few days during a school year owing to illness or other accepted causes, and this level of absence may be considered as nonproblematic and probably without adverse consequences.

Problematic SA has typically been differentiated in three main types: school refusal (SR), truancy (TR), and school withdrawal (SW). SR refers to SA related to emotional distress in the child, where the child does not try to hide absence from their parents, the child does not exhibit severe antisocial behavior, and the parents have made efforts to get their child to school. TR refers to SA related to externalizing problems, where the absence occurs without the permission of the school and the child typically tries to conceal the absence from their parents. SW refers to SA attributable to parental effort to keep the child at home or where there is little or no parental effort to get the child to school [1]. On the basis of their review of the conceptualization of problematic SA and the differentiation of school attendance problems (SAPs), Heyne et al. [1] concluded that although there is an overlap between the occurrence of SR and TR, between 83% and 95% of youth with problematic SA can be reliably classified as displaying SR, TR, or SW.

Interventions for SA have usually been designed for youths presenting with either TR or SR. A systematic review of TR interventions included 5 randomized controlled trials (RCTs) and 11 quasi-experimental design (QED) studies with a total of 1725 students [10]. Interventions aimed at improving school attendance were effective, overall, in reducing school SA with a moderate and significant mean effect size (*g* = 0.46; mean attendance improvement, 4.69 days). However, in 15 of the 16 studies the absence rates were still above 10% following intervention [10]. A recent systematic review of interventions for SR included six RCTs and two QED studies with a total of 425 students [11]. All but one study used a cognitive behavioral therapy (CBT) protocol. There was a moderate and significant mean effect size of attendance (*g* = 0.54). Findings from both reviews were based on a small number of studies and small sample sizes, and there was substantial heterogeneity between studies. Both reviews recommended conducting studies in which randomized controlled designs and larger sample sizes are used.

Most evidence-based treatments (EBTs) are single-disorder treatments and have been criticized for adapting poorly to the more complex and comorbid problems that are often seen in clinical practice [12], as well as in children with problematic SA. Owing to the heterogeneity of problematic SA, more comprehensive intervention approaches that incorporate treatment of both TR and SR are needed [10, 13, 14]. New transdiagnostic CBT interventions using a modular approach have been developed to target anxiety, depression, and behavior problems within the same manual. Weisz et al. conducted a large RCT using a modular CBT program targeting anxiety, depression, and conduct problems and compared it with TAU and standard EBTs. The results showed that the modular approach outperformed the other treatments on most clinical outcome measures [15]. Other transdiagnostic interventions have been developed and have been shown to be feasible for implementation in school settings [16]. In Denmark, a modular transdiagnostic CBT manual for treating anxiety, depression, and behavior problems (Mind My Mind [MMM]) has recently been developed [17] and is being tested in an RCT.

Some children with problematic SA display anxiety and/or depression; some display externalizing problems, some display both, and some display other problems, (e.g., at a family or school level). In addition, negative cognitions concerning the ability to cope with situations associated with school attendance have been shown to be prevalent among children with problematic SA [18, 19]. Self-efficacy concerning school situations has been found to increase following treatment, and treatment that increases self-efficacy may reduce anxiety, depression, and behavior problems and facilitate reengagement with schooling [20].

An intervention that addressed the needs of this very heterogeneous group therefore needs to be based on an initial assessment and case formulation, followed by a modular, transdiagnostic approach that includes evidence-based interventions for anxiety, depression, behavior problems, parent training and teacher training, and a focus on increasing self-efficacy.

The main objective of this study is to test the efficacy of Back2School (B2S) [21], a modular transdiagnostic CBT intervention aimed at increasing school attendance and decreasing anxiety, depression, and behavior problems among youth with problematic SA. The study uses an RCT design with an active control group receiving treatment as usual (TAU). Based on previous studies, our primary hypothesis is that the B2S intervention will be superior to TAU in improving school attendance. Secondary hypotheses are that the B2S intervention will be superior to TAU in reducing anxiety, depression, and behavior problems. We further hypothesize that improvement in school attendance will be mediated by reductions in the youths’ anxiety, depression, and behavior problems and increases in the youths’ and parents’ self-efficacy. Other members of our research team will perform an economic evaluation comparing the B2S group with the TAU group, both in terms of cost utility measured with a quality-of-life measure and in terms of cost benefit measured by subsequent obtained grades, youth education, employment, and income.

## Methods/design

### Study design

**Fig. 1:**
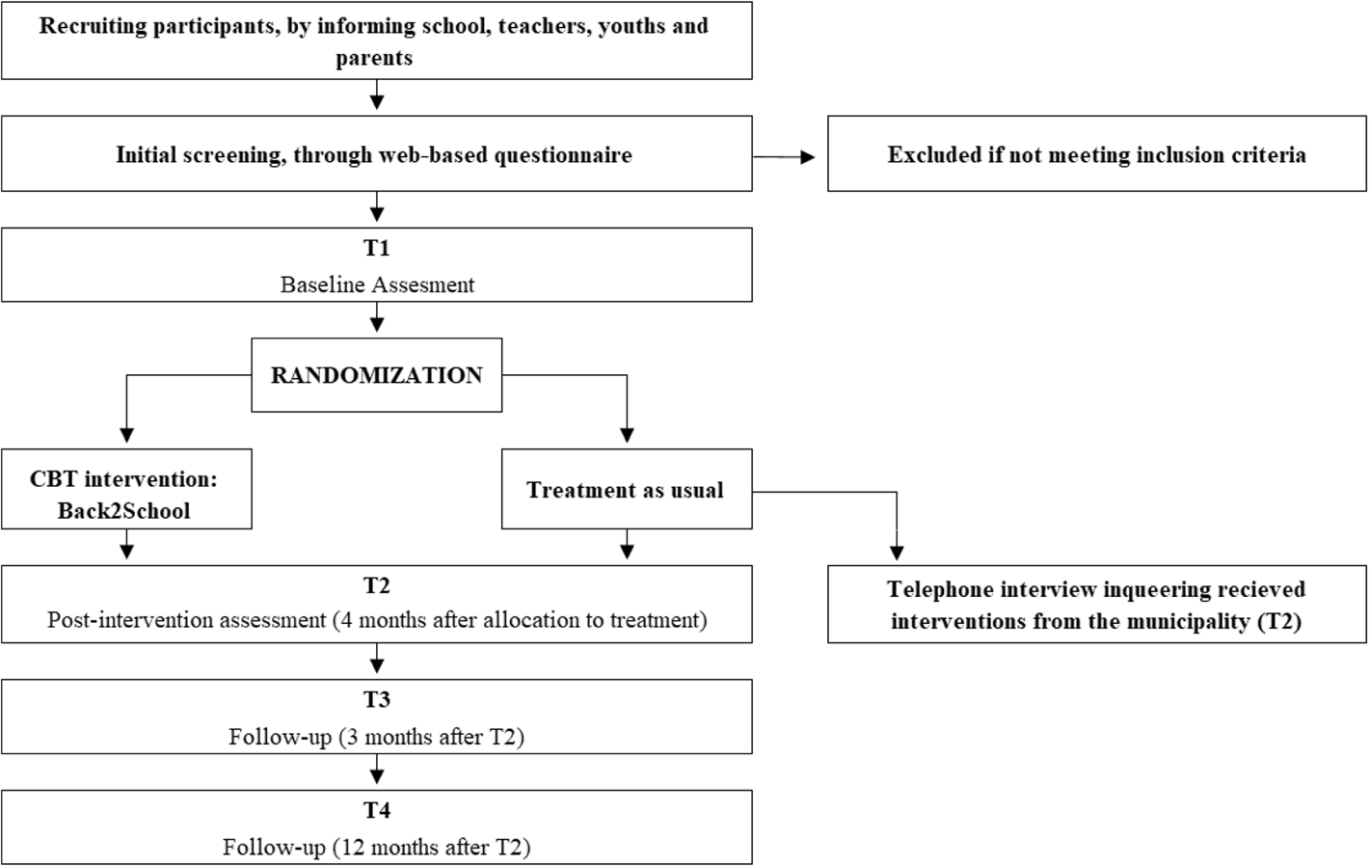
Flowchart of the Back2School study

The study is a randomized controlled, parallel group, superiority trial that compares TAU with a modular transdiagnostic CBT intervention (B2S) for SA in youths aged 7–16 years. The design is a two (Back2School and TAU) by four (preassessment [T1], postassessment [T2], and 3-month [T3] and 1-year [T4] assessments) mixed between-within design. The overall study design is illustrated in Fig. 1.

### Study setting

The study is a collaboration between Aarhus University and Aarhus Municipality, Denmark. The setting for both the B2S and TAU interventions is within Aarhus Municipality. The B2S intervention is developed and managed by the Center for Psychological Treatment for Children and Adolescents (CEBU) at Aarhus University and conducted at the same place. TAU interventions are conducted by Aarhus Municipality, and they take place at settings such as schools and social services within the municipality.

### Participants, recruitment, and eligibility criteria

Participants will be youth between 7 and 16 years old in primary and lower secondary school with a minimum of 10% parent-reported SA during the last 3 months. Because the study is conducted in collaboration with Aarhus Municipality, participants need to be registered at public schools in Aarhus Municipality. Private schools within Aarhus Municipality register students’ school absence differently from public schools, and they are outside the municipality’s jurisdiction, rendering school absence data unavailable. The study will include all youth from 0 to ninth grade, excluding participants in their second semester of ninth grade. The second semester of ninth grade is the final semester in Danish public schools, and after this semester, Aarhus municipality cannot provide absence data. Because we expect a larger attrition rate in the TAU group for the secondary measures, participants in the TAU group receive a shorter version of the postintervention assessment battery, and families are offered a gift card (value 200 DKK/26 EUR) after the completion of each subsequent assessment.

Participants are self-referred, and the families are required to make initial contact to participate in the study. They may be informed and directed by health or education professionals but cannot be formally referred. Prior to the start of the RCT, the municipality will implement extensive information and media campaigns aimed at families and professionals. Participants can contact project coordinators with questions within office hours via telephone or e-mail. The registration to participate will be through a web-based screening located at the B2S projects web page. The initial screening will be a short questionnaire based on inclusion criteria with the following questions: (1) language and school information, (2) parent-reported school absence regarding their child in the last 3 months (excluding holidays or other legal absence), and (3) contact information for one of the parents.

The study’s inclusion criteria are as follows: (1) enrolled in a public school within Aarhus Municipality; (2) aged 7–16 years and in 0–9th grade (excluding second semester of ninth grade); (3) report more than 10% SA during the last 3 months of school (based on parent-reported information); (4) the youth and at least one of the parents understand and speak Danish sufficiently to participate in treatment and complete questionnaires; (5) at least one of the parents is motivated to work on increasing the youth’s school attendance; (6) commitment to participate in assessment, intervention procedures, and acceptance of random assignment to intervention; and (7) written informed consent provided by the holders of the parental rights and responsibilities.

There are three main reasons for choosing the simple, low-threshold inclusion criteria of 10% absence during the last 3 months. First, the problems of SR and TR do not represent the full spectrum of youth with problematic SA. That is, these two types of absence are not exhaustive [22]. Basing the inclusion criteria on percentage of SA ensures that youth with other types of problematic SA are not excluded. Second, using a low threshold for absenteeism (only 10%) renders the results of the study more relevant to the broader population of youth with SAPs and not only to the smaller group of youth with severe SAPs (e.g., complete absence for the last 6 months). Third, the fact that parents are referring their children to the project for intervention suggests that parents perceive their child’s absence as problematic.

Participants who do not meet one or more of the inclusion criteria will be redirected in the online screening to a web page informing them of why they are not included in the study and where they can seek other help in the municipality. Participants passing the initial screening will receive verbal (by telephone) and written information and will provide informed consent by electronically signing a consent form. Families are informed that participation in the study is voluntary, that their consent can be withdrawn at any time, and that their participation or withdrawal from the study will not affect their access to the municipality’s usual support and treatment. Participating children and their parents will then receive the preintervention assessment battery, and it is required that the child and the parents complete all questionnaires. After completing the assessment battery, participants will be randomized to one of the treatment conditions within a maximum of 4 weeks. If the youth is randomized to participate in the B2S intervention, their main teacher will receive a preintervention assessment battery immediately after the randomization. All children and parents in both conditions, as well as the primary teacher in the B2S condition, will receive a postassessment battery and two follow-up assessment batteries. All assessment batteries are administered electronically.

### Randomization

Randomization to treatment condition will be conducted using a computer-generated random digit procedure with two possibilities (B2S and TAU). Treatment outcome of school absence may be affected by the age of participants and the amount of school absence. Therefore, to ensure balanced groups, the randomization will be stratified on the presence of two factors, *age* (first to fourth grade [younger] or fifth to ninth grade [older]) and *amount of school absence* (< 50% [low] or > 50% absence [high]). To maintain similar treatment group sizes, the randomization will be conducted using permuted block randomization. The randomization is administered by staff outside the research group.

### Intervention

#### Back2School program

B2S is a manualized CBT program developed for this study, aimed at treating youths with SA. The B2S program is used together with the transdiagnostic MMM manual [23]. The MMM manual comprises evidence-based CBT methods and techniques organized into disorder-specific modules to target subclinical or clinical levels of anxiety, depression, behavioral disturbance, and trauma-related problems. The CBT methods and techniques in the MMM manual are adapted from EBT programs targeting each of the specific domains of problems in children and adolescents. The MMM manual supplements the B2S program, and the B2S manual refers to relevant material from the MMM manual.

The B2S manual is specifically developed for treating SA. Intervention is determined via a descriptive functional analysis obtained via the School Refusal Assessment Scale (SRAS) [24] together with a case formulation approach to planning CBT for attendance problems. The functional approach involves identifying the motivational function of the child’s SA. Motivational functions include (1) avoidance of school-based situations that provoke negative affectivity, (2) avoidance of aversive school-based social/evaluative situations, (3) pursuit of attention from significant others outside of school, and (4) pursuit of tangible reinforcement outside of school [24–26]. The first two motivational functions refer to negative reinforcement; the latter two motivational functions refer to positive reinforcement. SA motivated by positive reinforcement suggests CBT procedures such as parent management, contingency management, and contracting to minimize incentives for SA and boost incentives for attendance. SA motivated by negative reinforcement suggests CBT procedures such as cognitive restructuring and exposure-based practice to reduce the anxious or depressive physical sensations and thoughts. In the development of the intervention, we adapted aspects of the @SCHOOL intervention [27] and the When Children Refuse School intervention [25, 28].

The intervention consists of a 1.5-h clinical interview with the youth and parents aimed at designing a case formulation and a treatment plan and preparing the family for the first therapy session, ten 1-h sessions with the child and parents together (except for sessions 2 and 6, which are only with the parents), a 1-h booster session with the child and parents together, and four school meetings. With the aim of instilling hope for change in the family, to speed up the change process, and to show the family that the SAP is taken seriously, the first 2 weeks of the intervention involve two sessions per week. For the following six sessions, there is the option to schedule them weekly or once every 2 weeks as decided to be appropriate by the therapist and the family together. The implementation of the booster session is flexible regarding the timing and will be held within 1–3 months after the last session. An important part of the B2S intervention is to collaborate with the school. In addition to the sessions with the child and parents, four meetings with participation of teachers from the youth’s school, the therapists, and the parents are conducted. The meetings will take place at the child’s school at the beginning, the middle, and the end of the treatment period, as well as shortly after the booster session. For a detailed overview of the intervention, *see* Table 1.

**Table 1:** Overview of the Back2School program

Session number	Duration (h)	Participants	Session content
S-0	1.5	T, C, P	Structured assessment interview with the family conducted by the therapists (a clinical psychologist and a clinical psychology graduate student). The family receive handouts regarding psychoeducation and SMART goals as homework for session 1.
Clinical conference	1	T	The therapists are discussing the case formulation, choice of treatment modules, and treatment goals with a clinical psychologist at CEBU
S-1	1	T, C, P	Presenting and discussing the case-formulation with the family. Psychoeducation regarding school absence, and development of SMART goals.
S-2	1	T, P	Parent only session 1. Helping the parents to clarify and solve potential questions/problems regarding school placement, somatic symptoms in child, and parental motivation for change. Planning better routines at home. Working with potential sleep problems.
S-3	1	T, C, P	Planning the date for returning to school, and planning the first day back in school. Creating a gradual exposure plan for returning to school.
S-4	1	T, C, P	Psychoeducation regarding the youth’s primary problem related to school absence (anxiety, depression, or behavioral problems) by including the MMM Modules. Continuing work with the gradual exposure plan for returning to school.
S-5	1	T, C, P	Continuing work with CBT methods regarding the youth’s primary problem related to school absence (e.g. exposure, behavioral activation and/or cognitive restructuring) by including the MMM Modules. Continuing work with the gradual exposure plan for returning to school. Working with boundaries.
S-6	1	T, P	Parent only session 2. Working with parent behavior. Identifying and reducing factors at home that maintain school absence.
S-7	1	T, C, P	Continuing to work towards returning to school. Revising gradual exposure plan. Focusing on how parents can support the youth in exposure exercises, and returning to school. Problem solving
S-8	1	T, C, P	Open session tailored to needs of the youth and parents. Continue working with CBT methods by including the MMM Modules. Open session tailored to needs of the youth and parents. Continue working with CBT methods by including the MMM Modules.
S-9	1	T, C, P	
S-10	1	T, C, P	Concluding the program. Focusing on maintaining and continuing the progress.
Booster	1	T, C,P	Focusing on maintaining and continuing the progress. Problem solving regarding relevant problems. Advise possible further help.
SM 1	1	T, P, S	Presenting and discussing the case formulation with the school. Planning the schools role in the youth’s return to school. Informing the school about the B2S and CBT approach.
SM 2	1	T, S	Following up on the youth’s progress in the school setting. Discussing potential academic difficulties, problems regarding bullying or other problems.
SM 3	1	T, S	Planning how the school can continue to help and support the youth. Discussing relapse prevention.
SM 4	1	T, S	Planning how the school can continue to help and support the youth. Discussing relapse prevention.

*Abbreviations: B2S* Back2School, *C* Child, *CBT* Cognitive behavioral therapy, *MMM* Mind My Mind, *P* Parent, *S* School officials, *S* Session, *SM* School meeting, *SMART* Specific, measurable, attainable, realistic, time-bound, *T* Therapist

#### Clinical interview and case formulation

Initially, the families in the B2S group attend a 1.5-h structured clinical interview held by the appointed therapists. The interview is designed to get an understanding of the youth’s SA, development, family and social situation, and functioning in daily life. The interview also includes a brief, semistructured psychopathological interview developed for the study with the child and parents together. Based on the qualitative and quantitative information derived from the interview and the preintervention assessment battery, a case formulation is developed by the therapists. At a clinical case conference, the case formulation is discussed with a clinical psychologist at CEBU, and a preliminary treatment plan is constructed.

#### Therapists

School psychologists from Aarhus Municipality and clinical psychologists from CEBU will conduct the B2S intervention together with a clinical psychology graduate student at CEBU as cotherapist. There is one psychologist and one cotherapist per case. All therapists and cotherapists receive a 6-day training course and four 1-day brush-up courses regarding assessment, case formulation, and the B2S and MMM manuals. In total, therapists and cotherapists receive 80 h of training. All therapists and cotherapists receive weekly face-to-face group case supervision by specialists in clinical child psychology.

### Treatment as usual

The help that the municipality provides to youths with SA varies and is dependent on the available resources in the school and the municipality, as well as the youths’ presenting problems. The TAU intervention is requested by the schools and is usually provided by Aarhus Municipality’s school psychologists, but it could also consist of counseling by teachers or social workers. For example, the interventions could be meetings with the school and/or the families, individual counseling with the child, flexible school hours, or transfer to special education classes (Aarhus Municipality, 2013). To keep track of the different interventions in the TAU condition, a telephone interview will be conducted with the parents in the TAU group at T2, investigating which interventions participants in the TAU condition have received.

### Outcomes

An overview of the included outcome measures and raters (child, parents, and teacher) is presented in Table 2.

**Table 2:** Overview of outcome measures, respondents, and assessment points

Measures	Respondent	Time							
		T1		T2		T3		T4	
		B2S	TAU	B2S	TAU	B2S	TAU	B2S	TAU
Primary outcome measure									
School absence: registry	M	●	●	●	●	●	●	●	●
School absence: parent-reported	P	●	●	●	●	●	●	●	●
Secondary outcome measures									
SDQ	Y, P, T	●	●	●	●	●	●	●	●
PECK	Y	●	●	●		●		●	
FAD	Y, P	●	●	●		●		●	
SCAS	Y, P	●	●	●		●		●	
MFQ	Y, P	●	●	●		●		●	
CHU-9D	Y	●	●	●	●	●	●	●	●
SEQ-SS	Y	●	●	●	●	●	●	●	●
SEQ-RSAP	P	●	●	●	●	●	●	●	●
Other measures:									
Background information	P, T	●	●	●	●	●	●	●	●
School and family collaboration	P, T	●	●	●	●	●	●	●	●
ESQ	Y, P, T			●	●				
SRAS-R	Y, P	●	●						

*Abbreviations: B2S* Back2School, *CHU-9D* Child Health Utility 9D Index, *ESQ* Experience of Service Questionnaire, *FAD* Family Assessment Device, *M* Aarhus Municipality, *MFQ* Mood and Feelings Questionnaire, *PECK* Personal Experience Checklist, *P* Parent, *SCAS* Spence Children’s Anxiety Scale, *SDQ* Strength and Difficulties Questionnaire, *SEQ-RSAP* Self-Efficacy Questionnaire for Responding to School Attendance Problems, *SEQ-SS* Self-Efficacy Questionnaire for School Situations, *SRAS-R* School Refusal Assessment Scale–Revised, *T* Teacher, *Y* Youth

#### Primary outcome

The primary outcome is school attendance, which is measured in two ways:

1. It is mandatory for all public schools in Denmark to report school absence data for all schoolchildren on a daily basis. Daily school absence data for youth included in the study will be provided by Aarhus Municipality. Absence data 1 year prior to the youths’ inclusion in the project and at follow-up are also provided by the municipality.
2. Retrospective daily school absence for a 2-week period (10 schooldays) is reported by parents at all assessment points (as part as the assessment battery at preassessment, postassessment, and follow-up).

In addition, the families in the B2S group will register daily absence for each lesson throughout their course in the B2S intervention.

#### Secondary outcomes

##### Strength and Difficulties Questionnaire

The Strength and Difficulties Questionnaire (SDQ) [29] will be used to measure emotional, behavioral, and social difficulties. The SDQ consists of a self-report version (from age 11) and two proxy report versions for parents and teachers. All three informants complete the SDQ. The SDQ is a brief behavioral screening questionnaire and consists of 25 items rated on a 3-point scale. The items are divided into five 5-item subscales that generate a score for emotional symptoms, conduct problems, hyperactivity/inattention, peer relationship problems, and prosocial behavior. The total difficulties scale sums up the difficulties across the four problem areas (not including lack of prosocial behavior). The extended version of the SDQ also asks questions about child distress and interference of problems with home life, friendships, classroom learning, and leisure activities, each scored on a 4-point scale. The impact scale sums up the distress and interference of problems, counting only the moderate and severe levels. The SDQ is a well-established and widely used measure that has shown good psychometric properties in a Danish population [30].

##### Spence Children’s Anxiety Scale

The Spence Children’s Anxiety Scale (SCAS) [31] is a self-report rating scale on which youths assess their symptoms of anxiety by answering 44 questions (including six positive filler items) on a 4-point scale. The scores are summed on six subscales reflecting symptoms specifically related to social phobia (six items), panic disorder and agoraphobia (nine items), generalized anxiety disorder (six items), obsessive-compulsive disorder (six items), separation anxiety disorder (six items), and fear of physical injury (five items). A total score reflects the overall severity of anxiety symptoms.

##### Parent version of the Spence Children’s Anxiety Scale

The parent version of the Spence Children’s Anxiety Scale (SCAS-P) [32] is a self-report rating scale on which parents assess their child’s symptoms of anxiety. It includes the same items as the SCAS but without the six filler items and is administered and scored like the SCAS. The Danish version of the SCAS and SCAS-P has demonstrated good psychometric properties [33].

##### Mood and Feelings Questionnaire

The Mood and Feelings Questionnaire (MFQ) [34] was developed to cover a broad range of cognitive and vegetative symptoms of depression in youths. The MFQ includes youth and parent versions (MFQ-P), consisting of 33 and 34 items, respectively, and each is rated on a 3-point scale. Studies show that the MFQ validly identifies children presenting with major depressive episodes, especially when the MFQ and the MFQ-P are used in combination. The Danish version of the MFQ has shown good psychometric properties [35].

##### Self-Efficacy Questionnaire for School Situations

The Self-Efficacy Questionnaire for School Situations (SEQ-SS) [18] was developed to assess the self-efficacy expectations of school-refusing youths. The SEQ-SS consists of 12 items and 2 subscales: academic/social stress and separation/discipline stress. Each item measures self-efficacy expectations related to different school situations on a 5-point scale. The total score is derived from summing the items together, yielding a total score. The SEQ-SS has been evaluated and shown to have good psychometric properties.

##### Self-Efficacy Questionnaire for Responding to School Attendance Problems

The Self-Efficacy Questionnaire for Responding to School Attendance Problems (SEQ-RSAP) (Heyne D, Maric M, Westenberg PM: Self-Efficacy Questionnaire for Responding to School Attendance Problems, Unpublished) has been developed to assess parents’ self-efficacy in relation to helping their child attend school regularly and without difficulty. The SEQ-RSAP consists of 13 items assessing parents’ self-efficacy for dealing calmly and constructively with the child’s difficulty attending school, rated on a 4-point scale. In a preliminary study of the psychometric properties of the SEQ-RSAP, the instrument showed promising convergent validity and good temporal stability (Lavooi M: Evaluation of the Self-Efficacy Questionnaire for Responding to School Attendance Problems, Unpublished).

##### Personal Experience Checklist

The Personal Experience Checklist (PECK) [36] was developed to provide a multidimensional assessment of youths’ personal experience of being bullied, covering a full range of bullying behaviors, including covert relational forms of bullying and cyberbullying. The youths are asked to rate on a 5-point scale how often they have experienced different forms of bullying over the last month, and the scale consists of 32 items and 4 subscales: relational-verbal bullying, cyberbullying, physical bullying, and bullying based on culture. An evaluation of the PECK scale has shown that it provides a promising assessment of a child’s experience of bullying behavior.

##### Family Assessment Device

The Family Assessment Device (FAD) [37] was designed to assess different dimensions of family function. It is rated by both youth (over the age of 12) and parents. It consists of 3 subscales with a total of 60 statements describing various aspects of family functioning. This study will use the subscale for general functioning (12 items). The FAD has been evaluated as a good measure of overall family functioning with good psychometric properties [38].

#### Collaboration between family and school

Collaboration between family and school will be rated by the schools and parents. This will be rated on three questions:

1. To what degree do you think that the cooperation between the school/teacher/family is working satisfactory?
2. To what degree do you think that the teacher/family listens your suggestions for change?
3. To what degree do you think that it is a good experience to talk to the teacher/family about your child/student?

These questions will be rated on a 4-point scale.

### Additional measures

#### Background information

Participating families will complete a background information questionnaire regarding family demographics, youth’s school and SA problems, youth’s mental and physical health, parents’ mental and physical health, and youth’s previous and ongoing treatment. Teachers complete information regarding the child’s academic function.

#### School Refusal Assessment Scale–Revised child version

The School Refusal Assessment Scale–Revised (SRAS-R) child version [39] was designed to evaluate the relative strength of four functional conditions of SR in youths: (1) avoid stimuli that provoke negative affectivity, (2) escape aversive social and/or evaluative situations, (3) pursue attention from significant others, and/or (4) pursue tangible reenforcers outside of school. The SRAS-R will be used as part of the assessment. The SRAS-R child version consists of a youth and parent version, both consisting of 24 items that are equally divided across the 4 functions and rated on a 7-point scale. The scale gives an indication of the strength of the four functional conditions of SR in the youths and is rated by both the youths and parents. The SRAS-R child and parent versions both have been shown to have good retest reliability and parent interrater reliability. A correlation between scores in SRAS-R child and parent versions has also been found.

#### Economic evaluation

The Child Health Utility 9D Index (CHU-9D) [40] was designed to determine how health affects children’s lives and is rated by the youth. The CHU-9D is a generic preference-based measure of health-related quality of life designed for the estimation of quality-adjusted life-years for economic evaluation of health care. It consists of nine dimensions (worry, sadness, pain, tiredness, annoyed feeling, schoolwork/homework, sleep, daily routine, and activities), each with five levels on which the child chooses the level fitting to how they are feeling. The instrument has previously been validated among children and adolescents in Great Britain and Australia, showing good psychometric properties [41, 42]**.** Socioeconomic data related to various background characteristics about children and parents and prospective data regarding grades, youth education, and employment will be extracted from Statistics Denmark’s registers and the registers of Aarhus Municipality and linked to survey data using the child’s civil registration number.

#### Treatment satisfaction

The revised version of the Experience of Service Questionnaire (ESQ), is used to assess satisfaction with the treatment [43]. The ESQ will be administered to youths, parents, and teachers at posttreatment (T2). There are separate versions for youths, with seven items, and parents and teachers, with ten items, including open questions for qualitative feedback.

#### Mediator measures

**Fig. 2:**
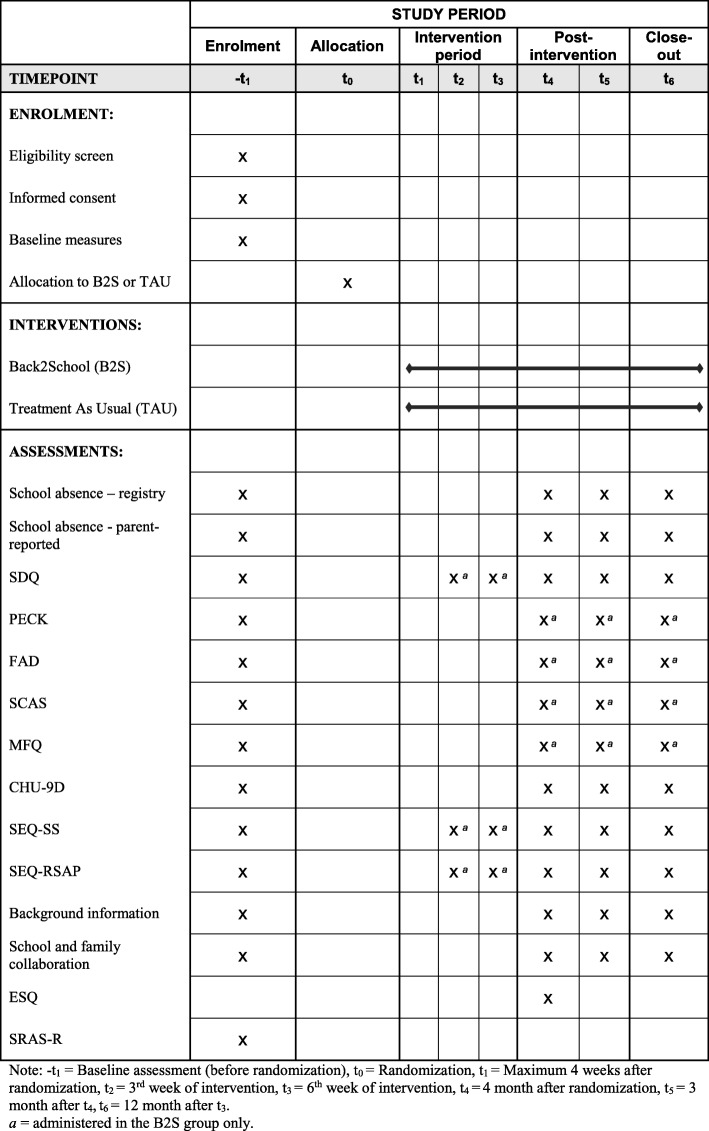
Standard Protocol Items: Recommendation for Interventional Trials (SPIRIT) diagram of schedule of enrollment, allocation, interventions, and assessments

As shown in Table 3, to investigate possible mediators for an increase in school attendance, the SDQ, the SEQ-SS, and the SEQ-RSAP will be administered at sessions 3 and 7 during the intervention in the B2S group. For an overview of the schedule of enrollment, allocation, interventions, and assessments, please *see* Fig. 2 for the completed Standard Protocol Items: Recommendation for Interventional Trials (SPIRIT) figure.

**Table 3:** Overview of mediator measures, and assessment points for participants in B2S condition

Measure	Respondent	Time	
		S-3	S-7
SDQ	Y, P	●	●
SEQ-SS	Y	●	●
SEQ-RSAP	P	●	●

*Abbreviations: P* Parent, *SCAS* Spence Children’s Anxiety Scale, *SDQ* Strength and Difficulties Questionnaire, *SEQ-RSAP* Self-Efficacy Questionnaire for Responding to School Attendance Problems, *SEQ-SS* Self-Efficacy Questionnaire for School Situations, *Y* Youth

### Sample size

On the basis of findings of recent meta-analyses of both truant SA [10] and SR SA [11], we expect to find a standardized effect size regarding SA in the range of 0.46–0.54. The targeted sample size is 70 per condition to provide sufficient statistical power (0.80) and a significance level (0.05, two-tailed) to find a generalized effect size regarding SA of 0.54. Similar RCTs have a mean attrition rate of 10% [44–47]; therefore, 80 participants are included in each condition (B2S *n* = 80, TAU *n* = 80).

### Statistical analysis

Analyses will be undertaken on an intention-to-treat basis. Any participants who are randomized but withdraw from the study will be included in the analysis as randomized.

#### Primary study parameters

Mixed linear models (MLMs) will be used to compare groups (B2S and TAU) over time (T1, T2, T3) for all recurrent outcome variables. Later, the same analyses will be performed for the follow-up period (T3, T4). MLMs will be used to measure main effects of group and time and the time × group interaction effects. MLMs tolerate missing values and thus do not unnecessarily compromise statistical power [48]. All MLMs will be estimated with the maximum likelihood method and based on the intention-to-treat sample. All models will include a random intercept, and the slope will be specified as random if improving the model fit evaluated by a significant change in the -2 log-likelihood (- 2LL) fit statistics [49]. A visual inspection of the data and an inspection of the model indices for the time variable will determine the best fit for the time variable. The outcomes of specific problems of relevance in the corresponding subgroups having anxiety, depression symptoms, or behavior problems as their primary problems will be explored.

#### Mediators

To test the hypothesis that the effects of the SA are mediated by the mediators investigated (i.e., internalizing and externalizing problems and self-efficacy), analytic steps outlined by MacKinnon et al. will be followed [50, 51].

## Discussion

Developing an effective intervention for children with SA is critically important because there are a great number of school-aged children who struggle to attend school regularly. The complex nature of SA is often handled with equally complex and unsystematic approaches. This makes it difficult for families to navigate and find the help that fits their situation and problems. There is a lack of systematic approaches for helping youths with SA, which can be tailored to fit the presenting problems of the youths and families that struggle with SA. The present study will provide information about the effectiveness of the manualized transdiagnostic multimodal CBT intervention B2S for treating SA. If the intervention is found to be efficacious, it could be a subject for large-scale implementation in school health services. The systematic program may be easier to implement by health professionals and provide better help for these youths and their families, but it needs to be compared with and found superior to the TAU intervention before such a conclusion can be drawn. In the present study, sound psychometric measures are used with multiple respondents in a study with an RCT design. The two conditions are studied with conditions that closely match a real-world setting.

### Trial status

A feasibility study of 24 children was performed in the spring of 2017, with high satisfaction scores and a low dropout rate. Based on the experiences from the feasibility study, the treatment manual and some of the procedures were revised. The present protocol is version 2, October 23, 2018. Inclusion of participants to the RCT started September 4, 2017. Inclusion is expected to be finished by September 4, 2019 (Additional file 1).

## Additional file

Additional file 1: Standard Protocol Items: Recommendation for Interventional Trials (SPIRIT) checklist. (DOCX 25 kb)

## Abbreviations

B2S: Back2School
CBT: Cognitive behavioral therapy
CEBU: Center for Psychological Treatment for Children and Adolescents
CHU-9D: Child Health Utility 9D Index
EBT: Evidence-based treatment
ESQ: Experience of Service Questionnaire
FAD: Family Assessment Device
M: Aarhus Municipality
MFQ: Mood and Feelings Questionnaire
MLM: Mixed linear model
MMM: Mind My Mind
P: Parent
PECK: Personal Experience Checklist
QED: Quasi-experimental design
RCT: Randomized controlled trial
SA: School absenteeism
SAP: School attendance problem
SCAS: Spence Children’s Anxiety Scale
SDQ: Strength and Difficulties Questionnaire
SEQ-RSAP: Self-Efficacy Questionnaire for Responding to School Attendance Problems
SEQ-SS: Self-Efficacy Questionnaire for School Situations
SR: School refusal
SRAS-R: School Refusal Assessment Scale–Revised
SW: School withdrawal
T: Teacher
TAU: Treatment as usual
TR: Truancy
Y: Youth

## References

[1] Heyne D, Gren-Landell M, Melvin G, Gentle-Genitty C. Differentiation between school attendance problems: why and how? Cogn Behav Pract. 2018; 10.1016/j.cbpra.2018.03.006.

[2] Egger HL, Costello EJ, Angold A (2003). School refusal and psychiatric disorders: a community study. J Am Acad Child Adolesc Psychiatry.

[3] Kearney CA (2008). School absenteeism and school refusal behavior in youth: a contemporary review. Clin Psychol Rev.

[4] McShane G, Walter G, Rey JM (2001). Characteristics of adolescents with school refusal. Aust N Z J Psychiatry.

[5] Baxter SD, Royer JA, Hardin JW, Guinn CH, Devlin CM (2011). The relationship of school absenteeism with body mass index, academic achievement, and socioeconomic status among fourth-grade children. J Sch Health.

[6] Carroll HCM (2010). The effect of pupil absenteeism on literacy and numeracy in the primary school. Sch Psychol Int.

[7] Evans LD (2000). Functional school refusal subtypes: anxiety, avoidance, and malingering. Psychol Schools.

[8] Hancock KJ, Shepherd CCJ, Lawrence D, Zubrick SR (2013). Student attendance and educational outcomes: every day counts.

[9] Undervisningsministeriet. Statistik om elevfravær. Copenhagen: Danish Ministry of Education; 2018. Retrieved September 8 from: https://www.uvm.dk/statistik/grundskolen/elever/elevfravaer.

[10] Maynard BR, Mccrea KT, Pigott TD, Kelly MS (2013). Indicated truancy interventions for chronic truant students: a Campbell systematic review. Res Soc Work Pract.

[11] Maynard BR, Heyne D, Brendel KE, Bulanda JJ, Thompson AM, Pigott TD (2018). Treatment for school refusal among children and adolescents: a systematic review and meta-analysis. Res Soc Work Pract.

[12] Chorpita BF, Weisz JR, Daleiden EL, Schoenwald SK, Palinkas LA, Miranda J (2013). Long-term outcomes for the Child STEPs randomized effectiveness trial: a comparison of modular and standard treatment designs with usual care. J Consult Clin Psychol.

[13] Kearney CA, Graczyk P (2014). A response to intervention model to promote school attendance and decrease school absenteeism. Child Youth Care Forum.

[14] Lyon AR, Cotler S (2009). Multi-systemic intervention for school refusal behavior: integrating approaches across disciplines. Adv School Ment Health Promot.

[15] Weisz JR, Chorpita BF, Palinkas LA, Schoenwald SK, Miranda J, Bearman SK (2012). Testing standard and modular designs for psychotherapy treating depression, anxiety, and conduct problems in youth: a randomized effectiveness trial. Arch Gen Psychiatry.

[16] Chu BC, Crocco ST, Esseling P, Areizaga MJ, Lindner AM, Skriner LC (2016). Transdiagnostic group behavioral activation and exposure therapy for youth anxiety and depression: initial randomized controlled trial. Behav Res Ther.

[17] Arendt K, Kjerholt C, Jeppesen P, Jørgensen L (2016). Mind My Mind Manual – træning af tanker, følelser og adfærd for skolebørn [Mind My Mind treatment Manual – training of thoughts, feelings and behaviour for schoolchildren].

[18] Heyne D, King N, Tonge B, Rollings S, Pritchard M, Young D (1998). The self-efficacy questionnaire for school situations: development and psychometric evaluation. Behav Chang.

[19] Maric M, Heyne DA, de Heus P, van Widenfelt BM, Westenberg PM (2012). The role of cognition in school refusal: an investigation of automatic thoughts and cognitive errors. Behav Cogn Psychother.

[20] Heyne DA, Sauter FM, Maynard BR, Maric M, Prins PJM, Ollendick TH (2015). Moderators and mediators of treatments for youth with school refusal or truancy. Moderators and mediators of youth treatment outcomes.

[21] Thastum M, Arendt K (2017). Back2School. Manual til behandling af børn med bekymrende skolefravær [Back2School. Manual for treatment of youth with problematic school absenteeism].

[22] Lyon AR, Cotler S (2007). Toward reduced bias and increased utility in the assessment of school refusal behavior: the case for diverse samples and evaluations of context. Psychol Sch.

[23] Jeppesen P. Transdiagnostic, Cognitive and Behavioral Intervention for in School-aged Children With Emotional and Behavioral Disturbances (MindMyMind RCT) (NCT03535805). (2018, May 24. Last updated 2018, May 28). Retrieved 2018, September 1, from https://clinicaltrials.gov/ct2/show/NCT03535805?term=jeppesen&rank=4:ClinicalTrials.gov.

[24] Kearney CA, Silverman WK (1993). Measuring the function of school refusal behavior - the School Refusal Assessment Scale. J Clin Child Psychol.

[25] Kearney CA, Albano AM (2007). When children refuse school: a cognitive-behavioral therapy approach: therapist guide.

[26] Kearney CA, Silverman WK (1996). The evolution and reconciliation of taxonomic strategies for school refusal behavior. Clin Psychol Sci Pract.

[27] Heyne D, Sauter FM, Ollendick TH, Van Widenfelt BM, Westenberg PM (2014). Developmentally sensitive cognitive behavioral therapy for adolescent school refusal: rationale and case illustration. Clin Child Fam Psychol Rev.

[28] Kearney CA, Albano AM (2007). When children refuse school: a cognitive-behavioral therapy approach: parent workbook.

[29] Goodman R (2001). Psychometric properties of the Strengths and Difficulties Questionnaire. J Am Acad Child Adolesc Psychiatry.

[30] Niclasen J, Teasdale TW, Andersen AM, Skovgaard AM, Elberling H, Obel C (2012). Psychometric properties of the Danish Strength and Difficulties Questionnaire: the SDQ assessed for more than 70,000 raters in four different cohorts. PLoS One.

[31] Spence SH (1998). A measure of anxiety symptoms among children. Behav Res Ther.

[32] Nauta MH, Scholing A, Rapee RM, Abbott M, Spence SH, Waters A (2004). A parent-report measure of children’s anxiety: psychometric properties and comparison with child-report in a clinic and normal sample. Behav Res Ther.

[33] Arendt K, Hougaard E, Thastum M (2014). Psychometric properties of the child and parent versions of Spence Children’s Anxiety Scale in a Danish community and clinical sample. J Anxiety Disord.

[34] Daviss WB, Birmaher B, Melhem NA, Axelson DA, Michaels SM, Brent DA (2006). Criterion validity of the Mood and Feelings Questionnaire for depressive episodes in clinic and non-clinic subjects. J Child Psychol Psychiatry.

[35] Eg J, Bilenberg N, Costello EJ, Wesselhoeft R (2018). Self- and parent-reported depressive symptoms rated by the Mood and Feelings Questionnaire. Psychiatry Res.

[36] Hunt C, Peters L, Rapee RM (2012). Development of a measure of the experience of being bullied in youth. Psychol Assess.

[37] Epstein NB, Bishop DS, Levin S (1978). The McMaster Model of Family Functioning. J Marriage Fam Couns.

[38] Miller IW, Ryan CE, Keitner GI, Bishop DS, Epstein NB (2000). The McMaster Approach to Families: theory, assessment, treatment and research. J Fam Ther.

[39] Kearney CA (2006). Confirmatory factor analysis of the School Refusal Assessment Scale–Revised: child and parent versions. J Psychopathol Behav Assess.

[40] Stevens K (2012). Valuation of the Child Health Utility 9D Index. PharmacoEconomics.

[41] Canaway AG, Frew EJ (2013). Measuring preference-based quality of life in children aged 6–7 years: a comparison of the performance of the CHU-9D and EQ-5D-Y—the WAVES pilot study. Qual Life Res.

[42] Furber G, Segal L (2015). The validity of the Child Health Utility instrument (CHU9D) as a routine outcome measure for use in child and adolescent mental health services. Health Qual Life Outcomes.

[43] Attride-Stirling J. Development of methods to capture users’ views of child and Adolescent mental health services in clinical governance reviews [updated 2002]. Retreaved 2018, September 1 from: https://www.corc.uk.net/media/1215/chi_projectevaluationreport.pdf.

[44] Heyne D, King NJ, Tonge BJ, Rollings S, Young D, Pritchard M (2002). Evaluation of child therapy and caregiver training in the treatment of school refusal. J Am Acad Child Adolesc Psychiatry.

[45] King NJ, Tonge BJ, Heyne D, Pritchard M, Rollings S, Young D (1998). Cognitive-behavioral treatment of school-refusing children: a controlled evaluation. J Am Acad Child Adolesc Psychiatry.

[46] Last CG, Hansen C, Franco N (1998). Cognitive-behavioral treatment of school phobia. J Am Acad Child Adolesc Psychiatry.

[47] Wu X, Liu F, Cai H, Huang L, Li Y, Mo ZJ (2013). Cognitive behaviour therapy combined fluoxetine treatment superior to cognitive behaviour therapy alone for school refusal. Int J Pharmacol.

[48] Twisk J, de Boer M, de Vente W, Heymans M (2013). Multiple imputation of missing values was not necessary before performing a longitudinal mixed-model analysis. J Clin Epidemiol.

[49] Heck RH, Thomas S, Tabata L (2013). Multilevel modeling of categorical outcomes using IBM SPSS.

[50] MacKinnon DP, Lockwood CM, Hoffman JM, West SG, Sheets V (2002). A comparison of methods to test mediation and other intervening variable effects. Psychol Methods.

[51] MacKinnon DP, Fairchild AJ, Fritz MS (2007). Mediation analysis. Annu Rev Psychol.

